# Supplementary material for: Causal interplay between lactose intolerance and gut microbiota: a combined bidirectional Mendelian randomization and in vivo validation study
Source: Front Nutr. 2026 Jun 1;13:1803337. doi: 10.3389/fnut.2026.1803337 (PMC13265576; doi:10.3389/fnut.2026.1803337)
Supplement: Supplementary file 1 [file Data_Sheet_1.zip › supplementary materials/Forward/leave one out cross validation/ebi-a-GCST90027772.finngen_R12_E4_LACTONAS.pdf]

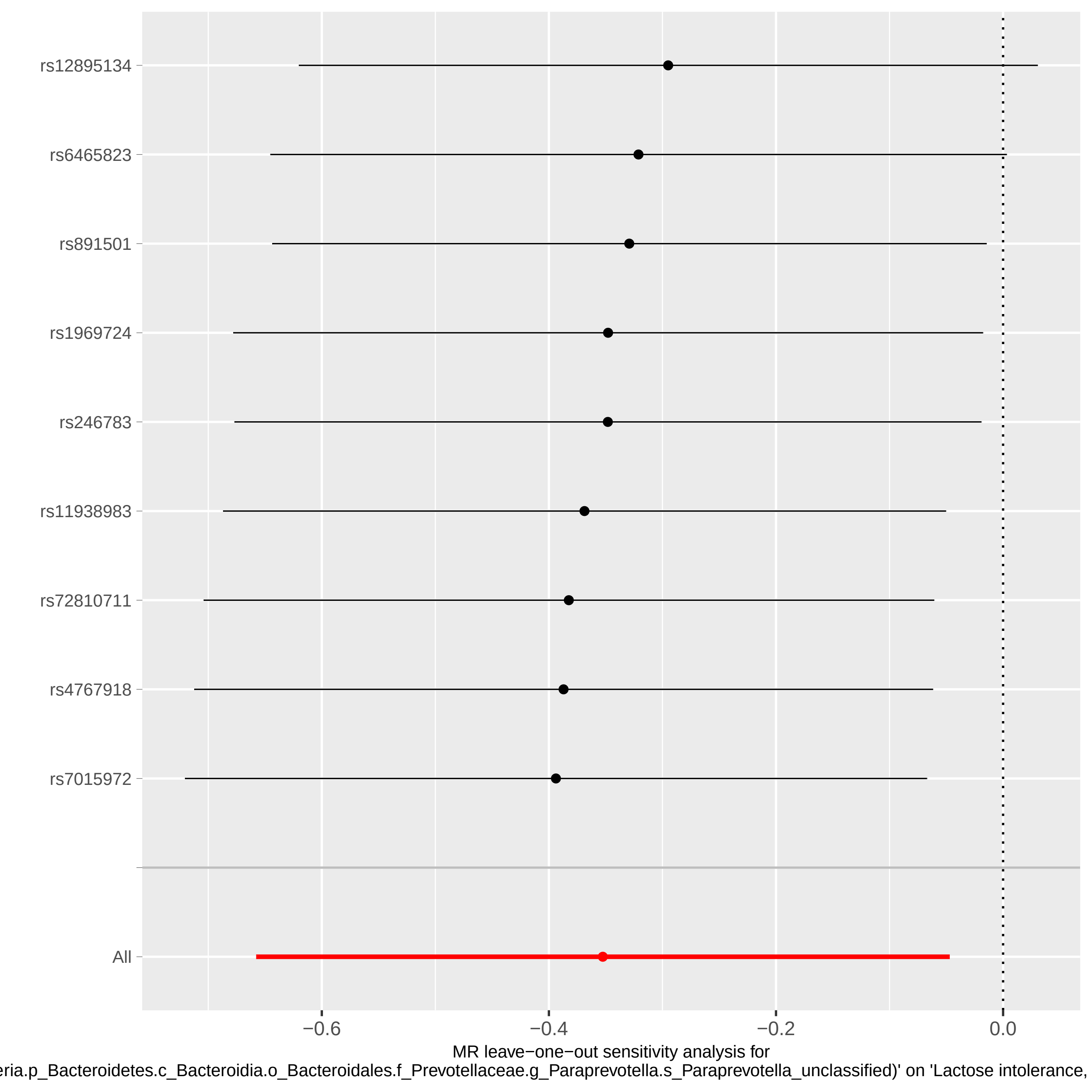
rs12895134
rs6465823
rs891501
rs1969724
rs246783
rs11938983
rs72810711
rs4767918
rs7015972
All
−0.6
−0.4
−0.2
0.0
MR leave−one−out sensitivity analysis for
eria.p_Bacteroidetes.c_Bacteroidia.o_Bacteroidales.f_Prevotellaceae.g_Paraprevotella.s_Paraprevotella_unclassified)' on 'Lactose intolerance,
